# Effectiveness of a behavioral medicine intervention in physical therapy on secondary psychological outcomes and health-related quality of life in exercise-based cardiac rehabilitation: a randomized, controlled trial

**DOI:** 10.1186/s13102-023-00647-x

**Published:** 2023-03-24

**Authors:** Sabina Borg, Birgitta Öberg, Lennart Nilsson, Joakim Alfredsson, Anne Söderlund, Maria Bäck

**Affiliations:** 1 Department of Health, Medicine and Caring Sciences, Unit of Physiotherapy, Linköping University, 581 83 Linköping, Sweden; 2 Department of Cardiology and Department of Health, Medicine and Caring Sciences, Linköping University, Linköping, Sweden; 3 Department of Health, Medicine and Caring Sciences, Unit of Cardiovascular Sciences, Linköping University, Linköping, Sweden; 4 Department of Physiotherapy, School of Health, Care and Social Welfare, Mälardalen University, Västerås, Sweden; 5 Department of Occupational Therapy and Physiotherapy, Sahlgrenska University Hospital, Gothenburg, Sweden

**Keywords:** Coronary artery disease, Control theory, Patient-reported outcome measurements, Secondary prevention, Self-efficacy

## Abstract

**Background:**

Interventions promoting adherence to exercise-based cardiac rehabilitation (exCR) are important to achieve positive physical and psychological outcomes, but knowledge of the added value of behavioral medicine interventions for these measures is limited. The aim of the study was to investigate the added value of a behavioral medicine intervention in physical therapy (BMIP) in routine exCR on psychological outcomes and health-related quality of life (HRQoL) versus routine exCR alone (RC).

**Methods:**

A total of 170 patients with coronary artery disease (136 men), mean age 62.3 ± 7.9 years, were randomized at a Swedish university hospital to a BMIP plus routine exCR or to RC for four months. The outcome assessments included HRQoL (SF-36, EQ-5D), anxiety and depression (HADS), patient enablement and self-efficacy and was performed at baseline, four and 12 months. Between-group differences were tested with an independent samples t-test and, for comparisons within groups, a paired t-test was used. An intention-to-treat and a per-protocol analysis were performed.

**Results:**

No significant differences in outcomes between the groups were shown between baseline and four months or between four and 12 months. Both groups improved in most SF-36 domains, EQ-VAS and HADS anxiety at the four-month follow-up and sufficient enablement remained at the 12-months follow-up.

**Conclusion:**

A BMIP added to routine exCR care had no significant effect on psychological outcomes and HRQoL compared with RC, but significant improvements in several measures were shown in both groups at the four-month follow-up. Since recruited participants showed a better psychological profile than the general coronary artery disease population, further studies on BMIP in exCR, tailored to meet individual needs in broader patient groups, are needed.

*Trial registration number* NCT02895451, 09/09/2016, retrospectively registered.

## Background

Depression and anxiety are common in patients with coronary artery disease (CAD) [1, 2] and are associated with an increased risk of mortality [1, 3] and reduced health-related quality of life (HRQoL) [4]. The benefits of exercise-based cardiac rehabilitation (exCR) for the secondary prevention of CAD are well established in clinical outcomes, such as reduced mortality and the risk of hospital readmission [5, 6], improved cardiovascular risk factor control [6] and aerobic exercise capacity [7]. ExCR has also shown positive effects on HRQoL [8, 9], anxiety and depression [10]. However, these patient-reported outcome measurements have been less studied in exCR than clinical outcomes.

Despite its proven efficacy, exCR remains widely underused [11]. At present, there is weak evidence of the effects of interventions aiming to increase adherence to exCR [12]. Behavioral medicine interventions have been used in a few studies of exCR to increase adherence to exercise programs and improve their physical and psychological outcomes [12–16]. Focht et al [16] reported favorable changes in HRQoL after participation in a behavioral medicine intervention within exCR [16]. Our present study used behavior change techniques based on control theory [17] to support an increase in self-efficacy for adherence to exCR [18]. Interventions using combinations of behavioral change techniques, derived from the control theory, such as self-monitoring, specific goal setting and feedback, have been shown to be effective in promoting exercise behavior in healthy adults [19]. These behavior change techniques have also shown to be positively associated with rehabilitation outcomes in patients with cardiac disease [13, 20].

The published main results [18], comparing exCR with or without a behavioral intervention in physical therapy (BMIP) added to routine care, showed significant intra-group improvements in exercise capacity for both groups at the end of the intervention. However, these changes did not differ significantly between the groups. In addition, improved exercise adherence was shown if a BMIP was added to exCR compared with routine exCR care alone (RC) [18]. Promoting adherence to exCR is important to improve positive health benefits [21]. However, more studies that evaluate the added value of behavioral medicine interventions in exCR on psychological outcomes and HRQoL are needed. The purpose of this pre-defined secondary analysis of the current study was to investigate the added value of a BMIP in routine exCR care on psychological outcomes and HRQoL versus routine exCR care alone.

## Methods

### Study design

This is an open-labeled, randomized, controlled trial.

### Participants

Patients were screened consecutively for study inclusion at a coronary care unit at a Swedish university hospital based on the following inclusion criteria: an index event due to type 1 myocardial infarction and/or percutaneous coronary intervention, age ≥ 18 years and < 75 years. The exclusion criteria were: the inability to understand the Swedish language and serious physical or mental health issues interfering with participation in exCR. Ethical approval was received from the Regional Ethical Review Board in Linköping, Sweden (registration number: 2015/209-31) and an amendment (registration number: 2018/383-32). Each participant provided informed written consent before entering the study. All methods were performed in accordance with the Declaration of Helsinki. The study is retrospectively registered at ClinicalTrials.gov (NCT02895451, 09/09/2016).

### Procedure

Physical therapists at the coronary care unit, received daily information about patients eligible for inclusion in the study and asked potential patients about participation. Baseline testing took place within two to three weeks after discharge. After finishing the baseline tests, the patients were randomized 1:1, using sealed, opaque envelopes, to a BMIP, combined with routine exCR care, or to routine exCR care alone (RC) for four months. Due to organizational reasons, the physical therapists performing the tests were not blinded to the intervention given. Three experienced physical therapists were responsible for the tests, and one experienced physical therapist, not involved in the testing procedure, was responsible for the BMIP intervention. The methods have been described in detail elsewhere [18, 22].

### Intervention

Table 1 describes behavior change techniques included in the present study and illustrates the differences in these behavior change techniques between BMIP and RC. The following behavior change techniques, based on the control theory, were used; prompt specific goal setting, prompt review of behavior goals, prompt self-monitoring of behavior, and the provision of feedback on performance [23]. The control theory describes a model of self-regulation [17] and is part of the Social Cognitive Theory of Self-Regulation [24] in which self-efficacy is important when it comes to changing a behavior [25]. Exercise adherence was defined as meeting at least 75% of the recommended exercise dose according to exCR guidelines [21, 26].

**Table 1:** Description of behavior change techniques used in the study

BCTs according to CT	Behavioral medicine intervention	Routine exCR care only
Specific goal setting	Detailed planning that specified what to do, the exercise dose and the context in which the exercise is to be performed	General planning regarding what to do, the exercise dose and the context in which the exercise is to be performed
	Action plan with strategies to promote possible facilitators and overcome barriers	No action plan was developed
	The patient’s motivation and self-efficacy for initiating and maintaining the exCR program were self-rated using a visual analogue scale and discussed in relation to the goals set	No self-rating or discussion about motivation and self-efficacy for initiating and maintaining the exCR in relation to the goals set
Review of behavior goals	The previously set exercise goals were comprehensively and structurally reviewed and/or reconsidered every third week during the intervention and at the end of the intervention	No structured review of exercise goals during the intervention. A less comprehensive and structural review at the end of the intervention
Self-monitoring of behavior	Patients kept a record of exercise in a diary, which was regularly followed up every three weeks during the exCR period	Patients kept a record of exercise in a diary, which was not followed up during the exCR period
Providing feedback on performance	Structured, comprehensive feedback every three weeks, including positive feedback on achieved goals, supporting feedback in relation to barriers, and the opportunity to discuss strategies to increase adherence	General feedback on performance during exercise sessions for patients participating in hospital-based exCR, and no feedback during the intervention for patients participating in a home-based setting
	Additional visual feedback on physical activity levels, using accelerometer data at one time during the intervention	No visual feedback on physical activity levels
	Structured follow-up meeting at the end of the intervention to discuss how the intervention had been perceived, goal achievement, long-term exercise goal setting, and potential future barriers, facilitators, and strategies	Less extensive structured and comprehensive follow-up meeting at the end of the intervention

BCT, behavior change technique; CT, control theory; exCR, exercise-based cardiac rehabilitation

#### Routine exCR care

The RC group followed routine exCR care. The exercise program was individually prescribed, based on tests of physical fitness, and was performed at the exCR center twice a week, under the supervision of a physical therapist. Each session included aerobic exercise for 20–60 min at an intensity of 40–85% of VO_2max_, corresponding to 12–17 according to Borg´s Rating of Perceived Exertion Scale, in combination with resistance exercise containing 8–10 different exercises, 10–15 repetitions in 1–3 sets [21, 26]. Patients were also instructed to perform one home-based aerobic exercise session/week, to achieve the recommendation of at least three aerobic exercise sessions/week. When preferred by patients, the choice to perform the exCR in a home-based setting was accepted. Three visits to a physical therapist for outcome assessment at baseline, four- and 12-months follow-up was part of the routine exCR care. Patients who performed the exCR program in a hospital-based setting also interacted with the physical therapist during the exercise sessions. Routine care did not include any structured intervention to control or increase adherence. However, since certain behavior change techniques, such as social support and verbal persuasion, already are included in routine exCR care, these were equal to all participants in the study. Furthermore, following routine exCR care, general goal setting for the exercise program were included. Patients also reported their home-based exercise sessions in an exercise diary, but no further feed-back or follow-up was provided in the RC arm.

#### Behavioral medicine intervention

##### Specific goal setting and re-evaluation of goals

The BMIP intervention began with a meeting with a physical therapist for detailed planning and specific goal setting for the exCR program including a discussion about motivation and self-efficacy. Facilitators and barriers, together with an appropriate action plan in relation to achieving the exercise goals, were identified. The exercise goals were re-evaluated both during and at the end of the intervention.

##### Self-monitoring and feedback

The performed exercise dose was self-monitored and documented in an exercise diary. The physical therapist gave feedback on the exercise diary every three weeks, comprising feedback on achieved goals, potential barriers, and the opportunity to discuss strategies to increase adherence. Visual feedback on physical activity levels, using accelerometer data, was also given at nine weeks. At the four-month follow-up, a meeting with the physical therapist to discuss goal achievement, intervention perception and long-term exercise goals took place.

To summarize, the behavioral medicine intervention included one meeting at baseline, four follow-ups during the intervention, one follow-up at the end of the intervention and one long-term follow-up at 12 months.

### Outcomes

Demographic and clinical patient characteristics were collected from patient interviews and medical records. Outcome assessment included the variables listed below and was performed at baseline, four and 12 months, except for patient enablement, which was measured at four and 12 months.

#### Health-related quality of life

The Short Form-36 (SF-36) comprises eight dimensions (physical functioning, role limitations due to physical problems, bodily pain, general health, vitality, social functioning, role limitations due to emotional problems and mental health), and two summary components, a physical component score and a mental component score. Items in each dimension are transformed into a score from 0 to 100, where a higher score indicates better health [27]. The EuroQoL 5 dimensions (EQ-5D) consists of five different aspects of health compiled to create an index score and a visual analog scale ranging from 0 (worst state of health) to 100 (best state of health) [28]. Both the SF-36 and the EQ-5D have been found to be reliable and valid for patients with CAD [29, 30].

#### Psychological outcomes

##### Anxiety and depression

The Hospital Anxiety and Depression Scale (HADS) consists of seven anxiety items and seven depression items from which separate anxiety (HADS-A) and depression (HADS-D) scores are calculated on a scale from 0 to 21. A cut-off score of ≥ 11 for definite cases of both HADS-A and HADS-D is recommended [31]. HADS has been shown to be reliable and valid for patients with CAD [32, 33] and lowering the cut-off score for definite cases of HADS-D from 11 to 8 has been found to improve the sensitivity of the instrument [33].

##### Self-efficacy

The Self Efficacy for Exercise Scale comprises nine situations that could affect participation in exercise, rated on a scale from 0 (not confident) to 10 (very confident), with a total score of 0–90. The Self Efficacy for Exercise Scale is considered reliable and valid for older adults [34] and for patients with CAD (Cavrak et al., unpublished data).

##### Patient enablement

The Patient Enablement Instrument consists of six questions, graded on a three-point scale ranging from 0 to 2, with a total score of 0–12, focusing on the ability to understand and cope with health issues and illness. A higher score indicates better patient enablement. The Patient Enablement Instrument has been found to be reliable and valid for patients in a primary care setting [35].

### Statistical methods

Sample size calculation has previously been reported in detail [18, 22]. Descriptive statistics were used for demographic data and are presented as the means and SD, or numbers and proportions (%), as appropriate.

Missing values were handled with multiple imputation. Patient characteristics and outcome measurements at baseline were included as independent variables, while outcome measurements at four and 12 months were entered as both independent and dependent variables in the multiple imputation model. The model used the chained equations procedure (fully conditional specification method in SPSS) to complete 10 data sets [36]. Constraints were applied according to the minimum and maximum value of each variable. Entering the EQ-5D index as a variable in the imputation model posed a problem and it was therefore not included in the analyses.

The within-group change, from baseline to four months and from four to 12 months, for each outcome was analyzed with paired-samples t-tests. Between-group differences, at each time point and the change between time points, were analyzed with an independent-samples t-test. The mean with 95% confidence intervals and *p*-values, based on the pooled results of each analysis, is presented.

A response analysis based on demographic data and outcome measurements at baseline was conducted, comparing responders and non-responders (participants with complete missing data) at the four-month follow-up. The response analysis revealed no substantial differences, whereby missing at random could be assumed. A statistical analysis was performed using SPSS statistical software for Windows (SPSS Version 25, IBM Corporation, New York, USA).

## Results

### Study population and demographic data

**Fig. 1:**
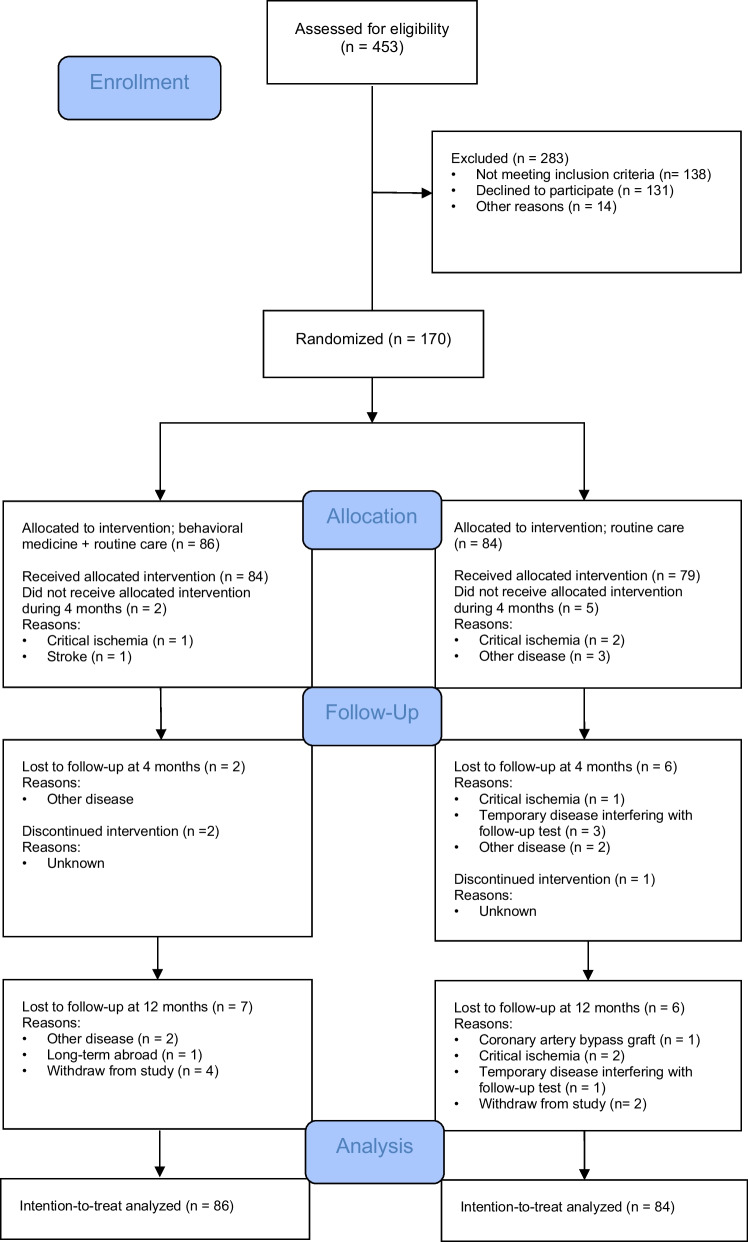
CONSORT flowchart of study participants

Of 453 patients, screened consecutively for study inclusion, a total of 170 patients (mean age 62.3 ± 7.9 years, 136 men) were recruited between January 2016 and October 2018. The study flowchart is presented in Fig. 1.

There was a significantly higher proportion of patients with unstable angina (*p* = 0.011) and a lower proportion of patients with non-ST-elevation myocardial infarction (*p* = 0.037), as the index event, in the BMIP group, compared to the RC group. No other significant differences in baseline demographics between the groups were found (Table 2).

**Table 2:** Baseline demographics for participants, n = 170

		Whole group (n = 170)	Behavioral medicine (n = 86)	Routine care (n = 84)
Age, years, mean ± SD		62.3 ± 7.9	62.7 ± 7.8	61.8 ± 8.0
BMI, kg/m^2^, mean ± SD		27.2 ± 4.1	27.2 ± 4.3	27.1 ± 4.0
Sex, n (%)	Male	136 (80)	72 (84)	64 (76)
	Female	34 (20)	14 (16)	20 (24)
Country of birth, n (%)	Sweden	165 (99)	86 (100)	79 (99)
	Other	1 (1)	0 (0)	1 (1)
Relationship status, n (%)	Married/partner	135 (79)	69 (80)	66 (79)
	Living alone	35 (21)	17 (20)	18 (21)
Educational level, n (%)	Elementary school	11 (7)	8 (9)	3 (4)
	High school	32 (19)	13 (15)	19 (23)
	Vocational school	44 (26)	20 (23)	24 (29)
	University	82 (49)	45 (52)	37 (45)
Occupational status, n (%)	Employed	89 (55)	43 (51)	46 (58)
	Retired	71 (44)	40 (48)	31 (39)
	Sick leave	2 (1)	0 (0)	2 (3)
	Unemployed, student, other	1 (1)	1 (1)	0 (0)
On sick leave at time of baseline assessment, n (%)		41 (45)	16 (36)	25 (52)
Smoking status, n (%)	Never smoked	73 (43)	36 (42)	37 (44)
	Ex- smoker > 1 month	83 (49)	45 (52)	38 (45)
	Smoker	14 (8)	5 (6)	9 (11)
Previous diseases, n (%)	MI	13 (8)	5 (6)	8 (10)
	PCI	18 (11)	8 (9)	10 (12)
	CABG	6 (4)	3 (4)	3 (4)
	Diabetes	17 (10)	11 (13)	6 (7)
	Hypertension	80 (47)	45 (52)	35 (42)
	Chronic heart failure	1 (1)	0 (0)	1 (1)
	Stroke	5 (3)	2 (2)	3 (4)
Type of index cardiac event, n (%)	STEMI	47 (28)	25 (29)	22 (26)
	NSTEMI	53 (31)	20 (23)	33 (39)
	Unstable angina	30 (18)	22 (26)	8 (10)
	Stable angina	40 (24)	19 (22)	21 (25)
Type of index cardiac intervention, n (%)	PCI	163 (96)	83 (97)	80 (95)
Left ventricular function, n (%)	Normal (EF > 50%)	93 (69)	53 (74)	40 (64)
	Lightly reduced (EF 40–49%)	28 (21)	13 (18)	15 (24)
	Moderate/severely reduced (EF < 40%)	14 (10)	6 (8)	8 (13)
Experience with exCR, n (%)		14 (8)	6 (7)	8 (10)

*BMI* Body mass index, *CABG* Coronary artery bypass graft, *EF* Ejection fraction, *exCR* exercise-based cardiac rehabilitation, *MI* Myocardial infarction, *NSTEMI* Non-ST-elevation myocardial infarction, *STEMI* ST-elevation-myocardial infarction, *PCI* Percutaneous coronary intervention

No statistical differences between the groups were found in HRQoL or psychological outcomes at baseline, except for a significantly higher SF-36 general health (*p* = 0.035) and a significantly lower HADS-A (*p* = 0.043) in the BMIP group compared with the RC group. The EQ-5D index showed a ceiling effect at baseline in both groups (BMIP: 0.895 (SD 0.12) and RC: 0.857 (SD 0.18)).

### Setting and adherence

The setting and adherence to the exCR program have previously been reported in detail [18]. Twenty patients in each group participated in hospital-based exCR, whereas 55 (BMIP) and 58 (RC) patients chose to perform the exCR in a home-based setting. Ten (BMIP) and 5 (RC) patients respectively participated in hospital-based exCR once a week in combination with home-based exercise. Twenty-three patients in the BMIP group and 13 patients in the RC group were defined as fully adherent to the exCR program [18]. This study had no serious adverse events related to exCR or the BMIP to report.

### Results of psychological outcomes and health-related quality of life

No significant differences between the groups were found for any of the outcomes between baseline and four months or between four and 12 months (Tables 3 and 4). Both groups improved significantly between baseline and four months in the EQ-VAS and in all SF-36 domains, except for bodily pain, mental health, and general health, and reported a significantly lower HADS-A. Significant improvements were also shown in the SF-36 general health in the RC group and in the SF-36; mental health in the BMIP group between baseline and four months. No significant difference in the change within groups between the four- and 12-month follow-ups were found, except for a significant decline in Self Efficacy for Exercise Scale in the BMIP group (Tables 3 and 4).

**Table 3:** Intention-to-treat analysis of change in health-related quality of life within and between groups

Variable			1. Routine care (n = 84)	2. Behavioral medicine (n = 86)	Between-group effects (1–2)
			Mean (95% CI), *p*-value	Mean (95% CI), *p*-value	Mean (95% CI), *p*-value
SF-36 PF	Baseline		86.2 (83.4–89.1)	87.0 (84.2–89.8)	− 0.7 (− 4.7–3.2), *p* = 0.711
	4 months		89.5 (86.8–92.2)	89.7 (87.4–92.0)	− 0.3 (− 3.9–3.4), *p* = 0.888
	1 year		89.2 (86.7–91.8)	90.5 (88.1–93.0)	− 1.3 (− 4.9–2.4), *p* = 0.498
	Within-group change	Baseline to 4 months	3.3 (0.7–5.8), *p* = 0.011	2.8 (0.2–5.3), *p* = 0.035	0.5 (− 3.1–4.1), *p* = 0.793
		4 months–1 year	− 0.2 (− 2.6–2.1), *p* = 0.835	0.8 (− 1.3–2.9), *p* = 0.476	− 1.0 (− 4.0–1.9), *p* = 0.504
SF-36 RP	Baseline		49.4 (40.3–58.5)	54.2 (45.3–63.1)	− 4.8 (− 17.3–7.8), *p* = 0.457
	4 months		78.5 (71.4–85.6)	81.4 (74.5–88.3)	− 2.9 (− 12.9–7.1), *p* = 0.573
	1 year		80.4 (73.5–87.3)	77.6 (70.3–84.9)	2.8 (− 8.1–13.7), *p* = 0.616
	Within-group change	Baseline to 4 months	29.1 (19.4–38.8), *p* < 0.001	27.2 (17.3–37.1), *p* < 0.001	1.9 (− 11.8–15.6), *p* = 0.787
		4 months–1 year	1.9 (− 6.5–10.3), *p* = 0.662	− 3.8 (− 13–5.4), *p* = 0.415	5.7 (− 6.5–17.9), *p* = 0.360
SF-36 BP	Baseline		75.2 (70.1–80.3)	75.8 (71.1–80.6)	− 0.6 (− 7.5–6.2), *p* = 0.854
	4 months		79.4 (74.5–84.3)	79.0 (73.9–84.2)	0.4 (− 7.4–8.1), *p* = 0.928
	1 year		73.9 (68.5–79.3)	77.6 (72.1–83.0)	− 3.7 (− 11.7–4.4), *p* = 0.376
	Within-group change	Baseline to 4 months	4.2 (− 1.7–10.2), *p* = 0.165	3.2 (− 3.0–9.4), *p* = 0.310	1.0 (− 7.8–9.8), *p* = 0.822
		4 months–1 year	− 5.5 (− 11.8–0.8), *p* = 0.091	− 1.5 (− 8.1–5.1), *p* = 0.661	− 4.0 (− 12.9–4.9), *p* = 0.377
SF-36 GH	Baseline		66.8 (62.2–71.5)	73.1 (69.6–76.7)	− 6.3 (− 12.1 to − 0.5), *p* = 0.035
	4 months		71.9 (67.7–76.0)	76.5 (72.2–80.7)	− 4.6 (− 10.6–1.4), *p* = 0.130
	1 year		71.7 (67.4–76.0)	74.0 (70.1–77.9)	− 2.3 (− 8.5–3.9), *p* = 0.465
	Within-group change	Baseline to 4 months	5.0 (1.4–8.6), *p* = 0.007	3.3 (− 0.3–7.0), *p* = 0.072	1.7 (− 3.3–6.7), *p* = 0.511
		4 months–1 year	− 0.1 (− 4.3–4.1), *p* = 0.955	− 2.4 (− 6.2–1.3), *p* = 0.206	2.3 (− 3.2–7.8), *p* = 0.407
SF-36 VT	Baseline		64.3 (59.5–69.2)	63.4 (58.9–68.0)	0.9 (− 5.7–7.5), *p* = 0.789
	4 months		69.6 (64.9–74.4)	71.8 (67.6–76.0)	− 2.1 (− 8.6–4.4), *p* = 0.526
	1 year		68.9 (64.7–73.0)	70.4 (66.3–74.6)	− 1.6 (− 8.0–4.8), *p* = 0.627
	Within-group change	Baseline to 4 months	5.3 (0.8–9.8), *p* = 0.021	8.3 (3.9–12.8), *p* < 0.001	− 3.0 (− 9.2–3.2), *p* = 0.341
		4 months–1 year	− 0.8 (− 4.7–3.1), *p* = 0.690	− 1.3 (− 5.5–2.9), *p* = 0.539	0.5 (− 4.8–5.9), *p* = 0.847
SF-36 SF	Baseline		83.8 (79.2–88.4)	85.0 (81.2–88.9)	− 1.2 (− 7.2–4.7), *p* = 0.680
	4 months		88.7 (84.7–92.8)	90.7 (86.9–94.5)	− 2.0 (− 7.7–3.8), *p* = 0.502
	1 year		89.0 (85.0–93.0)	93.0 (90.1–96.0)	− 4.0 (− 9.2–1.2), *p* = 0.130
	Within-group change	Baseline–4 months	5.0 (0.2–9.7), *p* = 0.040	5.7 (1.6–9.8), *p* = 0.007	− 0.7 (− 6.9–5.5), *p* = 0.822
		4 months to 1 year	0.3 (− 4.5–5.1), *p* = 0.912	2.3 (− 1.7–6.3), *p* = 0.255	− 2.1 (− 7.8–3.7), *p* = 0.482
SF-36 RE	Baseline		65.8 (56.7–74.9)	74.4 (66.2–82.7)	− 8.6 (− 20.7–3.5), *p* = 0.166
	4 months		83.4 (76.9–89.9)	86.2 (80.0–92.4)	− 2.8 (− 12.1–6.6), *p* = 0.566
	1 year		84.7 (78.2–91.1)	85.8 (79.8–91.7)	− 1.1 (− 10.2–8.0), *p* = 0.814
	Within-group change	Baseline to 4 months	17.6 (8.4–26.8), *p* < 0.001	11.7 (2.9–20.6), *p* = 0.010	5.8 (− 6.8–18.5), *p* = 0.365
		4 months–1 year	1.3 (− 7.8–10.4), *p* = 0.783	− 0.4 (− 8–7.2), *p* = 0.920	1.7 (− 9.3–12.7), *p* = 0.767
SF-36 MH	Baseline		76.8 (72.6–80.9)	79.6 (76–83.3)	− 2.9 (− 8.3–2.6), *p* = 0.301
	4 months		80.9 (77.5–84.2)	85.8 (82.7–89)	− 4.9 (− 10.3–0.4), *p* = 0.071
	1 year		82.0 (78.7–85.3)	86.2 (83.2–89.2)	− 4.2 (− 8.9–0.6), *p* = 0.084
	Within-group change	Baseline to 4 months	4.1 (− 0.4–8.6), *p* = 0.074	6.2 (2.9–9.5), *p* < 0.001	− 2.1 (− 7.8–3.6), *p* = 0.472
		4 months–1 year	1.1 (− 2.5–4.7), *p* = 0.545	0.3 (− 3.1–3.7), *p* = 0.842	0.8 (− 4.4–5.9), *p* = 0.770
SF-36 PCS	Baseline		46.3 (44.7–47.9)	46.9 (45.3–48.5)	− 0.6 (− 2.8–1.7), *p* = 0.615
	4 months		49.8 (48.1–51.4)	49.9 (48.2–51.6)	− 0.1 (− 2.6–2.3), *p* = 0.927
	1 year		49.0 (47.2–50.8)	49.1 (47.6–50.7)	− 0.1 (− 2.7–2.4), *p* = 0.923
	Within-group change	Baseline to 4 months	3.5 (1.8–5.2), *p* < 0.001	3.0 (0.8–5.3), *p* = 0.008	0.5 (− 2.2–3.2), *p* = 0.738
		4 months–1 year	− 0.8 (− 2.7–1.2), *p* = 0.437	− 0.8 (− 2.8–1.3), *p* = 0.460	0.0 (− 2.6–2.6), *p* = 0.993
SF-36 MCS	Baseline		46.3 (43.6–48.9)	48.0 (45.8–50.3)	− 1.8 (− 5.2–1.7), *p* = 0.317
	4 months		49.5 (47.4–51.6)	51.5 (49.6–53.4)	− 2.0 (− 5.0–1.0), *p* = 0.187
	1 year		50.1 (48.2–52.0)	51.8 (50.1–53.6)	− 1.8 (− 4.4–0.9), *p* = 0.190
	Within-group change	Baseline to 4 months	3.2 (0.7–5.7), *p* = 0.013	3.5 (1.2–5.7), *p* = 0.003	− 0.3 (− 3.6–3.0), *p* = 0.873
		4 months–1 year	0.6 (− 1.6–2.8), *p* = 0.590	0.3 (− 1.6–2.3), *p* = 0.735	0.3 (− 2.5–3.1), *p* = 0.855
EQ-VAS	Baseline		75.9 (72.3–79.4)	77.9 (75.0–80.7)	− 2.0 (− 6.5–2.5), *p* = 0.388
	4 months		80.2 (77.4–82.9)	81.7 (79.0–84.4)	− 1.5 (− 5.6–2.6), *p* = 0.465
	1 year		80.2 (77.6–82.8)	81.0 (78.3–83.8)	− 0.8 (− 5.0–3.3), *p* = 0.698
	Within-group change	Baseline to 4 months	4.3 (0.5–8.1), *p* = 0.028	3.8 (0.6–7.0), *p* = 0.019	0.5 (− 4.4–5.3), *p* = 0.853
		4 months–1 year	0.0 (− 2.9–3.0), *p* = 0.981	− 0.7 (− 3.7–2.4), *p* = 0.666	0.7 (− 3.5–4.9), *p* = 0.742

*BP* Bodily pain, *CI* Confidence interval, *EQ-VAS* EuroQoL visual analogue scale, *GH* General health, *MCS* Mental component score, *MH* Mental health, *PCS* Physical component score, *PF* Physical functioning, *RE* Role limitations due to emotional problems, *RP* Role limitations due to physical problems, *SF* Social functioning, *SF-36* Short form-36, *VT* Vitality

**Table 4:** Intention-to-treat analysis of change in psychological outcomes within and between groups

Variable			1. Routine care (n = 84)	2. Behavioral medicine (n = 86)	Between-group effects (1–2)
			Mean (95% CI), *p*-value	Mean (95% CI), *p*-value	Mean (95% CI), *p*-value
HADS anxiety	Baseline		4.4 (3.6–5.1)	3.3 (2.7–4.0)	1.0 (0.0–2.0), *p* = 0.043
	4 months		3.0 (2.4–3.6)	2.7 (2.0–3.4)	0.3 (− 0.6–1.3), *p* = 0.532
	1 year		3.3 (2.6–4.0)	2.9 (2.2–3.5)	0.4 (− 0.6–1.5), *p* = 0.415
	Within-group change	Baseline to 4 months	− 1.4 (− 2.0 to − 0.8), *p* < 0.001	− 0.7 (− 1.1 to − 0.2), *p* = 0.011	− 0.7 (− 1.5–0.1), *p* = 0.077
		4 months–1 year	0.3 (− 0.3–0.9), *p* = 0.334	0.2 (− 0.3–0.6), *p* = 0.505	0.1 (− 0.7–0.9), *p* = 0.756
HADS depression	Baseline		2.2 (1.7–2.7)	2.0 (1.5–2.5)	0.2 (− 0.5–0.9), *p* = 0.605
	4 months		2.2 (1.7–2.8)	1.7 (1.2–2.2)	0.5 (− 0.2–1.3), *p* = 0.173
	1 year		2.1 (1.6–2.5)	1.9 (1.5–2.3)	0.2 (− 0.5–0.9), *p* = 0.561
	Within-group change	Baseline to 4 months	0.1 (− 0.4–0.6), *p* = 0.827	− 0.3 (− 0.7–0.1), *p* = 0.193	0.3 (− 0.3–1.0), *p* = 0.326
		4 months–1 year	− 0.2 (− 0.7–0.4), *p* = 0.547	0.2 (− 0.2–0.5), *p* = 0.354	− 0.3 (− 0.9–0.3), *p* = 0.319
Self-efficacy for exercise scale	Baseline		51.2 (46–56.5)	57.7 (52.6–62.8)	− 6.5 (− 13.7–0.8), *p* = 0.080
	4 months		49.7 (44.5–54.8)	56.5 (51.6–61.4)	− 6.8 (− 14–0.3), *p* = 0.062
	1 year		46.6 (41.7–51.5)	50.6 (45.5–55.8)	− 4.0 (− 11.1–3.1), *p* = 0.266
	Within-group change	Baseline to 4 months	− 1.6 (− 7.6–4.5), *p* = 0.615	− 1.2 (− 6.4–4), *p* = 0.650	− 0.3 (− 8.2–7.6), *p* = 0.932
		4 months–1 year	− 3.1 (− 7.9–1.8), *p* = 0.219	− 5.9 (− 10.8 to − 1), *p* = 0.020	2.8 (− 3.9–9.5), *p* = 0.409
Patient enablement instrument	4 months		6.0 (5.2–6.7)	6.8 (6.1–7.6)	− 0.9 (− 2.1–0.3), *p* = 0.148
	1 year		5.9 (5.2–6.7)	6.2 (5.4–6.9)	− 0.3 (− 1.4–0.9), *p* = 0.658

*CI* Confidence interval, *HADS* Hospital anxiety and depression scale

## Discussion

This study contributes to previous research by presenting the effects of a BMIP added to routine exCR care on psychological outcomes and HRQoL versus routine exCR care alone. The four-month follow-up showed significant improvements in most SF-36 domains, EQ-VAS and HADS-A for both groups, but no significant differences in outcomes between the groups were found between baseline and four months or between four and 12 months.

The lack of differences between the groups in psychological outcomes and HRQoL after exCR in the present study, irrespective of an addition of a BMIP, confirm the previously reported challenges of trying to achieve a behavioural change to exercise in patients as part of work to enhance rehabilitation outcomes [37–40]. The inability to detect differences between the groups in the current study, is also in line with a recent Cochrane review, concluding that no significant differences between the groups for theory-based interventions that aimed to increase adherence in a CR-setting was found [12]. Increasing adherence to exCR is important since this will improve the effect of the treatment [41]. The current BMIP was intended to increase adherence to the exCR program and to assess the benefits of exercise on psychological outcomes and HRQoL. As previously reported, although adherence was higher in the BMIP group (31%) compared with RC (19%), it was lower than expected which may be one factor to explain the non-significant differences between groups [18].

Another possible explanation for the lack of group differences in the current study is the selection of included patients. Partcipating patients in the present study reported better HRQoL compared with previously published studies in patients with CAD [42–44]. Moreover, in comparison with the general Swedish population, the baseline EQ-5D index was higher in our study [45], in contrast to a European study reporting a lower EQ-VAS in patients with CAD compared with a general population [42]. For the SF-36, the baseline values in our study were higher compared with previously reported reference values in patients with CAD [44] but lower compared with the general Swedish population [46]. In terms of anxiety and depression, patients in the current study reported lower baseline values as compared to a previous cardiac population and a reference population in a study by Hansen et al. [47]. This means that healthy patients with CAD with a potentially greater interest in their own health are more likely to attend this kind of study. This may lead to selection bias and the risk of underestimating possible effects of the intervention, since the room for improvement is reduced by already favorable values at baseline.

Despite this, we found significant improvements for both groups in most SF-36 domains, EQ-VAS and HADS-A at the four-month follow-up. Our results are consistent with two recently published meta-analyses, showing significant improvements in multiple SF-36 domain scores after exCR [8, 9]. Focht et al. [16] reported favorable changes in domains of SF-36 in a behavioral medicine intervention group and in men in a routine exCR group, compared with women in the routine exCR group, with the greatest improvement in patients with low baseline values [16]. A recently published European position statement describes a 10% improvement in HRQoL and anxiety/depression score after participation in exCR as a relevant quality indicator for CR [48]. However, due to the favorable baseline values in our study, the potential for an improvement of this kind in all outcomes was limited. Furthermore, consideration also needs to be taken to the fact that HRQoL and psychological outcomes typically improves by clinical course after an index cardiac event, irrespectively of intervention given [49–51].

Except for a significant decline in Self Efficacy for Exercise Scale in the BMIP group between the four- and 12-month follow-ups, no significant difference within or between the groups was found regarding self-efficacy for exercise. Probably this could be due to high levels of self-efficacy for exercise in both groups at baseline. In line with this, a previous study reported the highest level of self-efficacy at the beginning of the CR programme [52]. Cederbom et al. [53] found significant improvements in self-efficacy for exercise within a behavioral medicine intervention group for older women compared with receiving regular physical activity advice, however, consistent with the results of the present study, no significant difference in self-efficacy between the groups was found [53].

Patient enablement represents patients’ empowerment and ability to cope, understand, and manage with their illness [54], and has to our knowledge not previously been reported in patients with CAD. The Patient Enablement Instrument values illustrating the self-rated effect on enablement after the intervention at four months showed levels similar to the previously reported median values after treatment for other diagnostic groups [55]. A Patient Enablement Instrument value of ≥ 6 has been reported to be relevant as an indication of meaningful effect in studies of primary care [56]. The Patient Enablement Instrument values in the current study are consistent for both groups at the 12-month follow-up, showing that adequate enablement remains. This aspect is important for patients´ active role in their treatment, which in turn may have a positive impact on adherence [41].

The lack of group differences in the current study could also be explained by the design of the BMIP and the fact that certain elements of behavior change techniques, such as social support, are already included in routine exCR care. The importance of social support from healthcare providers, peers and family members has been found to be one of the most frequently described factors influencing patients participation in exCR [57, 58] and were equal to all participants in the present study. Behavior change techniques used in the present BMIP included structured and comprehensive goal setting, self-monitoring and feedback. However, general goal setting for the exercise program, some elements of general feedback and keeping record of exercise in a diary, were also provided to patients within routine exCR care. Furthermore, it was difficult to completely control for what education, support, and encouragement physical therapists and other caregivers within the comprehensive CR program gave to patients in both groups. Consequenly, it is possible that both groups were provided with enough support, and that the added value of the current BMIP, was not sufficient to make a difference between the groups.

The settings where exCR was delivered could also possibly have an impact on the results since different settings include different possibilities to provide behavior change techniques. Advantages of supervised exercise programs include access to knowledge, feedback, and support from the healthcare provider during the exercise sessions [41] as well as vicarious experiences from the group-based exCR, together with social support received by peers [59]. Unsupervised exercise programs can, on the other hand, provide increased flexibility as they can be performed whenever the patient wishes without having to adapt to specific exercise schedules [41]. In the present study, however, the proportion of patients in the different settings was equally distributed in both groups with a majority of patients taking part in a home-based setting. Behavior change techniques such as goal setting, self-monitoring and social support from family and friends have been expressed as important aspects for continued exercise after the end of an exCR program [60], while lack of support and guidance from the healthcare provider has been stated as a factor that made the maintenance of exercise more challenging [59, 60]. Supervised exercise includes advantages in possibilities to provide behavior change support during the exercise sessions, which may increase self-efficacy for the exercise program [41]. Exercise as being adaptable to the environment of the patient, has on the other hand been described as a facilitator in home-based settings [61] and may in this sense involve a minor change in the transition to maintain the exercise behavior after the completion of a phase 2 exCR program. The possible long-term effects of a BMIP in exCR need to be further explored.

In the current study, the same content of the BMIP was given to all participants and tailoring to meet indivudal needs was limited in the context of a randomized controlled trial. Nor was it possible to adapt and tailor the design of the BMIP based on aspects associated with non-attendance at exCR in an individual, such as low self-efficacy and depression [62]. The present study included a selection of patients with a better psychological profile compared with the general CAD population, highlighting the need to investigate the effect of a BMIP in broader patient groups and to use psychological screening of each patient to adapt and tailor interventions based on individual needs.

### Strengths and limitations

We used a randomized, controlled design with a long-term follow-up, which is a strength when it comes to assessing the effectiveness of treatments. Reliable and validated questionnaires were used for collecting outcome data. The behavior change techniques are grounded in a theoretical framework [17], shown to be positively associated with rehabilitation outcomes in interventions in both healthy adults [19] and in patients with cardiac disease [13, 20]. Moreover, since the intervention does not require any additional education, it is easy to implement in the existing routine exCR care. The study population was mainly representative of typical Swedish exCR programs [63] which affects the generalizability of the results. On the other hand, the patients included reported better HRQoL and psychological outcome measures compared with previously published studies [42, 43, 47], and were motivated to take part in an exCR program. The sample size calculation was performed on the primary outcome in the current study and not on psychological outcomes and HRQoL which is a limitation in the study as well as the use of a single-center design. Due to organizational circumstances, physical therapists could not be blinded to the group allocation of patients. However, group allocation was kept confidential in relation to patients and the test procedure was validated by the involved physical therapists.

## Conclusions

A BMIP added to routine exCR care showed no significant difference in effects on psychological outcomes and HRQoL compared with routine exCR care alone. Despite favorable baseline values, both groups improved significantly in multiple domains of the SF-36, EQ-VAS and HADS-A after completing the exCR program and sufficient enablement remained at the 12-month follow-up. There is still room for further development of BMIP in exCR, including greater tailoring to individual needs in a more heterogenous population of patients with CAD, but this needs to be investigated in future studies.

## Data Availability

The datasets generated and/or analyzed during the current study are not publicly available due to identifying patient data should not be shared but are available from the corresponding author on reasonable request.

## Abbreviations

BMIP: Behavioral medicine intervention in physical therapy
CAD: Coronary artery disease
EQ-5D: EuroQol 5 dimensions
exCR: Exercise-based cardiac rehabilitation
HADS: Hospital anxiety and depression scale
HADS-A: Anxiety subscale of HADS
HADS-D: Depression subscale of HADS
HRQoL: Health-related quality of life
RC: Routine care
SD: Standard deviation
SF-36: Short-form 36
VAS: Visual analogue scale
